# A phase I clinical trial of dose escalation of lobaplatin in combination with fixed-dose docetaxel for the treatment of human solid tumours that had progressed following chemotherapy

**DOI:** 10.3892/ol.2014.2675

**Published:** 2014-11-05

**Authors:** YU PENG, YUE-E LIU, XIAO-CAN REN, XUE-JI CHEN, HUI-LING SU, JIE ZONG, ZENG-LI FENG, DONG-YING WANG, QIANG LIN, XIAN-SHU GAO

**Affiliations:** 1 Hebei North University, Zhangjiakou, Hebei 075000, P.R. China; 2 Department of Oncology, North China Petroleum Bureau General Hospital of Hebei Medical University, Renqiu, Hebei 062552, P.R. China; 3 Department of Radiation Oncology, Peking University First Hospital, Xicheng, Beijing 100034, P.R. China

**Keywords:** dose escalation, lobaplatin, docetaxel, chemotherapy, neoplasm

## Abstract

In this study, the maximum tolerated dose (MTD) of lobaplatin (LBP) when it was combined with docetaxel (TXT) for the treatment of solid tumours that had progressed following chemotherapy was determined, and toxicities to this regimen were evaluated. A modified Fibonacci method was used for the dose escalation of LBP. The patients received TXT (at a fixed dose of 60 mg/m^2^) on day one (d1) and LBP (at an initial tested dose of 30 mg/m^2^) on day two (d2) of a treatment cycle that was repeated every 21 days. Each dose group consisted of at least three cases. In the absence of dose-limiting toxicity (DLT), we proceeded to the next dose group, with a dose increment of 5 mg/m^2^ between groups, until DLT occurred. The dose immediately below the dose that produced DLT was regarded as the MTD. The 17 patients examined in this study completed a total of 58 cycles of chemotherapy, and a total of three dose-escalation groups (30 mg/m^2^ LBP, 35 mg/m^2^ LBP, and 40 mg/m^2^ LBP) were established. The main adverse event that was observed was myelosuppression. DLT occurred in four patients, including three patients in the 40 mg/m^2^ LBP group and one patient in the 35 mg/m^2^ LBP group. In total, three out of the four patients in the 40 mg/m^2^ LBP group exhibited DLT. We determined that the treatment administered to the 35 mg/m^2^ LBP group represented the MTD. Thus, our phase I trial revealed that the MTD for the tested LBP combination regimen was 35 mg/m^2^ LBP and 60 mg/m^2^ TXT. This regimen resulted in mild adverse reactions and favourable patient tolerance. Therefore, we recommend the use of these dosages in phase II clinical trials.

## Introduction

Statistics indicate that the incidence of cancer in China is increasing each year (1). Moreover, the clinical diagnosis of many Chinese patients occurs when these individuals have late-stage cancer and therefore no longer have the opportunity to receive radical surgical treatment. Chemotherapy has become an important method of cancer treatment, however, most patients undergo either relapse or metastasis following first-line chemotherapy, requiring second-line and subsequent treatments.

Cisplatin (DDP)-based chemotherapy is significant in cancer treatment; however, due to its toxicity, particularly nephro- and neuro-toxicity, DDP-based chemotherapy has limited applications (2,3). Therefore, researchers have pursued a new platinum compound. Lobaplatin (LBP), which is a class III platinum anticancer drug developed by the German firm ASTA Medica (Degussa), is primarily used for the treatment of advanced breast cancer, small cell lung cancer (SCLC), and chronic myeloid leukaemia (4). Research has demonstrated that LBP has various advantageous properties, including strong anticancer activity, no significant nephrotoxicity or neurotoxicity, no requirement for hydration/liquid infusion (5), a much lower incidence of drug resistance than DDP, and no cross-resistance with DDP (6,7). The mechanism of action of LBP is similar to that of other platinum drugs; in particular, LBP induces the formation of inter-strand Pt-GG and Pt-AG crosslinks, blocking DNA replication and transcription and thereby inhibiting gene expression in tumour cells (6). LBP’s pharmacokinetic characteristics include the rapid onset of clinical effects, the persistence of these effects for a long duration, high tumour tissue concentrations, and low plasma concentrations (8). Thus, the drug demonstrates good selectivity after entering the body. LBP exhibits superior pharmacokinetic parameters in Chinese populations compared with Western populations (8,9). A number of studies have revealed that LBP has broad-spectrum anticancer activity, including efficacy against lung cancer, breast cancer, colorectal cancer, testicular cancer, and lymphoma (2,10,11).

Docetaxel (TXT) is a semisynthetic compound in the taxane class of anti-cancer drugs. It binds to free tubulin, promotes the assembly of tubulin into stable microtubules, and inhibits microtubule depolymerisation (12). These effects significantly decrease the quantities of free tubulin and thereby inhibit cell mitosis and proliferation. Single-agent chemotherapy with TXT is an important treatment approach for a variety of tumours (13–16). Studies have demonstrated that the administration of LBP in combination with TXT can produce certain therapeutic effects in patients with tumour progression after chemotherapy and that in this combination, LBP and TXT produce synergistic effects (11). However, the optimal LBP dose in this combination regimen has not been established based on the findings from phase I/II clinical trials. In particular, although international studies have established a recommended LBP dose of 50 mg/m^2^ for single-agent chemotherapy (17–20), no phase I studies on the appropriate LBP dose in the aforementioned combination regimen for second-line or third-line chemotherapy have been reported. In Europe and the USA, the recommended dose of TXT for second-line therapy is 75–100 mg/m^2^ (21), however, Asian studies have suggested that a TXT dose of 60 mg/m^2^ is more suitable for East Asian populations (22–24). Our previous studies have demonstrated that Eastern and Western populations have different tolerances for the same doses of chemotherapy (cisplatin with 5-fluorouracil, and capecitabine with docetaxel), with tolerated doses in the combination regimens for Eastern populations that are equivalent to 70% to 80% of the corresponding doses for Western populations (25,26). Therefore, it is unclear whether chemotherapy doses determined based on studies of Western populations can be applied to Chinese patients. To further investigate the appropriate LBP dose in the aforementioned combination regimen, we conducted a dose escalation trial for LBP in this regimen; this study reports the results of this trial.

## Materials and methods

### Eligibility

The patients who participated in this study were pathologically or cytologically confirmed to have advanced solid tumours that had progressed after at least first-line chemotherapy. These patients had at least one evaluable lesion (27,28) and were in clinical stages III or IV. The following inclusion criteria were utilised: 18–75 years of age; Karnofsky Performance Status (KPS) score ≥60, with expected survival of over three months; a complete recovery to normal from the toxicity of prior treatment, with ≥four weeks since any previous treatment; marrow conditions that included a white blood cell (WBC) count, ≥4.0×10^9^/l, neutrophils, ≥1.5×10^9^/l, platelets (PLT), ≥100×10^9^/l, and haemoglobin, ≥100 g/l; adequate hepatic and renal function (serum creatinine, aspartate aminotransferase, alanine aminotransferase and total serum bilirubin ≤upper limits of normal); normal cardiopulmonary function, with no obvious infection, gastrointestinal bleeding, or other serious visceral diseases; no prior treatment with LBP; no treatment with TXT during the previous six months; favourable compliance to the chemotherapy regimen during the study period; and the provision of written informed consent.

### Exclusion criteria

Pregnant or lactating women; patients with no self-awareness, uncontrollable central nervous system metastases, uncontrollable seizures, or mental illnesses impairing self-awareness or judgment; patients who had been treated with chemotherapy drugs other than LBP and TXT or with radiation therapy within the prior four weeks; patients with organ transplants; and patients with the long-term use of immunosuppressive agents and corticosteroids.

### Pretreatment evaluation

Within one week prior to the start of treatment, the researchers obtained the subjects’ medical histories and KPS scores as well as completing a physical examination, a routine blood examination, tests of liver and renal function, chest and abdominal computed tomography (CT) imaging, and an electrocardiogram for each study subject.

### Trial Design

The trial was an open-label, non-randomised dose escalation study. Each group consisted of at least three patients. The primary endpoint of this study was to determine the maximum tolerated dose (MTD) of LBP in a LBP and TXT combination regimen for the treatment of solid tumours with progression following chemotherapy. The secondary endpoint of the study was to evaluate the safety, toxicity, and time to progression (TTP) of this LBP and TXT combination regimen.

### Ethics

The study was approved by the Ethics Committee, North China Petroleum Bureau General Hospital of Hebei Medical University, Renqiu, China. It was performed in accordance with the ethics standards of human experimentation and with the Helsinki Declaration of 1975, as revised in 2000. All patients provided written informed consent.

### Chemotherapy

A fixed dose of 60 mg/m^2^ TXT was diluted in 250 ml of 5% glucose and then intravenously injected on day one (d1) (22–24). LBP was dissolved in 5 ml of injectable sterile water, diluted in 250 ml of 5% glucose, and then intravenously injected in a 2 h treatment on day two (d2). A prophylactic anti-allergy treatment of 8 mg of dexamethasone was administered twice per day on the three consecutive days of d-1, d1, and d2. This treatment cycle was repeated every 21 days for a minimum of two cycles. The treatment was continued until disease progression or unacceptable toxicity was observed, up to a maximum of six cycles of chemotherapy.

During therapy, all patients were given 5-hydroxytryptamine (HT3) receptor antagonists as an anti-emetic prophylactic treatment. To ensure the continuity of chemotherapy, at WBC <4.0×10^9^/l and/or absolute neutrophil count (ANC) <2.0×10^9^/l, supportive treatment using recombinant human granulocyte colony-stimulating factor was administered, and when PLT <75×10^9^/l, interleukin-11 treatment was administered. If clinically indicated, additional supportive care was allowed.

### Dose escalation and the determination of dose-limiting toxicity (DLT)

The evaluation of adverse events was based on the Common Terminology Criteria for Adverse Events, v3.0 (29). DLT was defined to be the occurrence of one or more of the following events after the first day of chemotherapy and prior to the third cycle of chemotherapy: i) Haematological toxicity in the form of grade IV neutropenia, grade III febrile neutropenia, grade III or grade IV thrombocytopenia, or grade III or grade IV anaemia; ii) grade III–IV non-haematological toxicity (with the exception of alopecia, nausea, vomiting, and fatigue); and iii) any grade V responses.

A modified Fibonacci method (30) was used, with an initial LBP dose of 30 mg/m^2^ and a subsequent increase of 5 mg/m^2^ from one group to the next. The patients received the treatment specified by the study protocol in accordance with their order of enrolment, and the study gradually progressed from enrolling subjects in a low-dose group to enrolling subjects in a high-dose group. At least three patients were enrolled in each group. If DLT did not occur in the three cases in a dose group, the subsequent dose group was initiated. However, repeated administration to the same patient was not allowed. If one case of DLT occurred within a dose group, three additional patients were enrolled into the dose group in question. Enrolment into the subsequent dose group could only commence if none of these additional three patients experienced DLT. If one or more cases of DLT occurred among these additional three patients, the trial was terminated. The dose used in the final group was regarded as the dose that produced DLT, and the dose immediately below the dose that produced DLT was regarded as the MDT.

### Evaluation Standards

We used RECIST (Response Evaluation Criteria in Solid Tumours) 1.1 to evaluate short-term efficacy (31). The time point at which the efficacy evaluation was performed was one week prior to the third chemotherapy cycle. The following classifications were used for this evaluation: complete remission (CR), partial response (PR), stable disease (SD), and progressive disease (PD). The response rate (RR) was defined to be CR + PR, and the disease control rate (DCR) was defined to be CR + PR + SD. The main image-based evidence used for these evaluations consisted of the CT/magnetic resonance imaging (MRI) results. It was thought that with the exception of cases involving PD, no evaluations of efficacy could be performed after only one cycle of chemotherapy. Each patient received at least two cycles of chemotherapy. The chemotherapy cycle was repeated every 21 days, and treatment was continued until the occurrence of disease progression or unacceptable toxicity.

### Subsequent treatment

Based on the judgements of the research team, patients with disease progression received either third-line treatment or the best available supportive care.

### Follow-up

Following the completion of the treatment, the patients underwent follow-up studies once per month for the first six months and once every three months subsequently. Each follow-up exam included the acquisition of a medical history, a physical examination, a routine blood examination, comprehensive biochemical tests, and a chest CT; in addition, an abdominal CT was performed once every three months. All patients were followed up by outpatient examinations and telephone, and follow-up continued until mortality occurred.

### Statistical analysis

Data analysis was performed using SPSS 18.0, and the Kaplan-Meier method was used to calculate patients’ TTP.

## Results

### Patient characteristics

Between May 2012 and November 2013, 17 patients with malignant solid tumours were enrolled in this study. These patients included nine males and eight females, and their ages ranged from 45–76 years (with a median age of 62 years). The median KPS score of the study participants was 80 points (with a range of 60–90). Patients’ body surface areas ranged from 1.41–1.94 m^2^ (median, 1.66 m^2^). There were 11 cases of non-small cell lung cancer (NSCLC), two cases of SCLC, two cases of breast cancer, one case of gastric cancer, and one case of endometrial carcinoma. There was one stage IIIA patient, two stage IIIB patients, and 14 stage IV patients. A total of 14 patients among the entire group of study subjects were evaluable for efficacy; all 17 patients were evaluable for toxicity (Table I). Until 5th January 2014, no cases had been lost to follow-up, and the follow-up rate was 100%.

### Compliance

A total of 17 patients completed a total of 58 cycles of chemotherapy. The median number of chemotherapy cycles completed by a patient was four (range, 1–6 cycles). Two patients completed one chemotherapy cycle, six patients completed two chemotherapy cycles, five patients completed four chemotherapy cycles, and four patients completed six chemotherapy cycles.

### Haematological toxicity

Table II describes the haematological toxicities associated with the tested treatments. The incidence of leukopenia was 82.35% (11 cases), the incidence of grade III leukopenia was 29.41% (five cases), and there were no cases of grade IV leukopenia. The incidence of neutropenia was 58.82% (ten cases), the incidence of grade III neutropenia was 29.41% (five cases), and the incidence of grade IV neutropenia was 11.76% (two cases). The incidence of anaemia was 58.82% (ten cases), with no cases of grade III or grade IV anaemia. The incidence of thrombocytopenia was 46.2% (six cases), the incidence of grade III thrombocytopenia was 5.88% (one case in the 40 mg/m^2^ group), and there were no cases of grade IV thrombocytopenia.

### Non-haematological toxicity

The subjects experienced only mild non-haematological toxicities of grades I–II. There were no treatment-related deaths. There was one case of diarrhoea (5.88%), one case of phlebitis (5.88%), five cases of fatigue (29.41%), three cases of nausea (17.65%), and no cases of vomiting. All the patients improved after receiving symptomatic treatment. The adverse events are detailed in Table III.

### Determination of MTD

DLT did not occur in the chemotherapy group that received the initial LBP dose of 30 mg/m^2^, which was administered to the first three enrolled patients. In accordance with the dose escalation method, three patients were then enrolled into the next highest dose group, the 35 mg/m^2^ LBP group; as previously, DLT did not occur. Subsequently, four patients were enrolled into the next dose group (40 mg/m^2^ LBP group); the first of these patients withdrew from the trial on the seventh day after the first cycle of chemotherapy due to associated pancreatitis. We hypothesise that this occurrence of pancreatitis, which caused the patient to succumb to the disease seven days following the onset, was not directly caused by the chemotherapy. This is due to the fact that a search of the literature did not identify any studies which reported that lobaplatin or docetaxel could induce pancreatitis, and pancreatitis was not mentioned as a side effect in the instructions for the two drugs. DLT occurred in the second patient. This patient experienced grade III thrombocytopenia with a PLT of 26×10^9^/L on the 11^th^ day after one cycle of chemotherapy and succumbed to the disease on the day 12 following chemotherapy due to massive haemoptysis. This patient, who received only a single cycle of chemotherapy, exhibited central lung cancer with T4 lesions. On the third day following chemotherapy, pain and numbness in the patient’s left arm were significantly reduced, and swelling on the left side of the patient’s face had subsided significantly, suggesting that the chemotherapy was effective. Massive haemoptysis may have occurred due to damage to major blood vessels caused by rapid tumour regression (32); therefore, this patient’s outcome was not considered to be a case of chemotherapy-related death. The third patient in the 40 mg/m^2^ LBP group experienced grade IV neutropenia (a case of DLT), and the fourth patient in this group experienced grade III febrile neutropenia (a case of DLT). Therefore, at the third dose level, two patients each completed two cycles of chemotherapy, and experienced DLT (in the form of neutropenia), and one patient completed one cycle of chemotherapy, but also experienced DLT (in the form of grade III thrombocytopenia). We determined that the 40 mg/m^2^ level of LBP was the dose that produced DLT, and dose escalation was therefore terminated. To further evaluate the adverse events associated with 35 mg/m^2^ LBP, three additional patients were enrolled into the LBP 35 mg/m^2^ group. Among these patients, one case of DLT (in the form of grade IV neutropenia) was observed. Subsequently, an additional four patients were enrolled into this group, however, no other cases of DLT were observed. Therefore, in the 35 mg/m^2^ group, there were a total of 10 patients, and one case of DLT was observed. The patient who experienced DLT exhibited lung cancer with multiple bone metastases. This patient’s poor bone marrow function may have contributed to the onset of grade IV neutropenia. The patient’s neutrophil levels returned to normal after seven days, and no febrile neutropenia was observed. The aforementioned observations suggested that the 35 mg/m^2^ dose level was well tolerated; therefore, we do not consider this dose to be regularly associated with DLT. In summary, this study had three dose levels, as indicated in Table IV. Initially, three patients were enrolled in the dose group I, which consisted of 30 mg/m^2^ LBP on d2 and 60 mg/m^2^ TXT on d1, and DLT did not occur. In total, 10 patients were enrolled in dose group II, which consisted of 35 mg/m^2^ LBP on d2 and 60 mg/m^2^ TXT on d1, and only one patient experienced DLT. In contrast, four patients were enrolled in dose group III, which consisted of 40 mg/m^2^ LBP on d2 and 60 mg/m^2^ TXT on d1, however, one patient withdrew from the group, and the other three patients experienced DLT. These results suggested that in the tested regimen, the dose level of 40 mg/m^2^ LBP was overly strong and was not tolerated by the patients. In accordance with our experimental design, we determined that the MTD was the highest tested dose less than the dose that produced DLT; therefore, the MTD for the tested regimen was 35 mg/m^2^ LBP on d2 and 60 mg/m^2^ TXT on d1 of a treatment cycle that repeated every 21 days.

### Short-term efficacy

Among the 17 examined patients, 14 patients were evaluable. Among these 14 patients, there were no cases of CR, one case of PR, 10 cases of SD, and three cases of PD. Thus, the RR was 7.1% (1/14), and the DCR was 78.6% (11/14). Among the 11 examined NSCLC patients, nine patients were evaluable. These patients included one case of PR, six cases of SD, and two cases of PD. Thus, among the NSCLC patients, the RR was 11.1% (1/9), and the DCR was 77.8 % (7/9).

### Survival analysis

**Figure 1:**
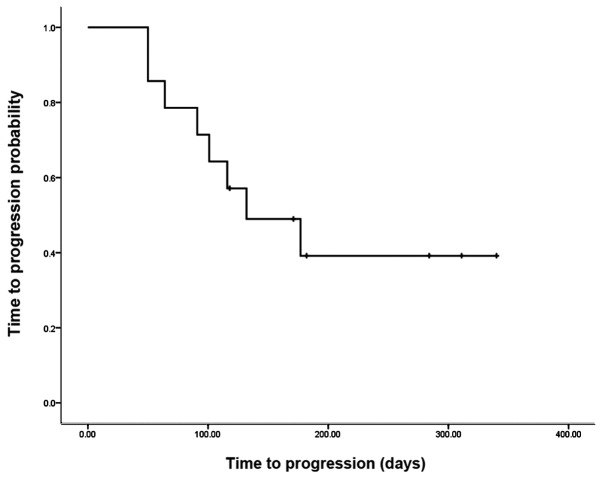
TTP for all patients: The median TTP was 132 days, and 6-months TTP rate was 39.2%. TTP, time to progression.

**Figure 2:**
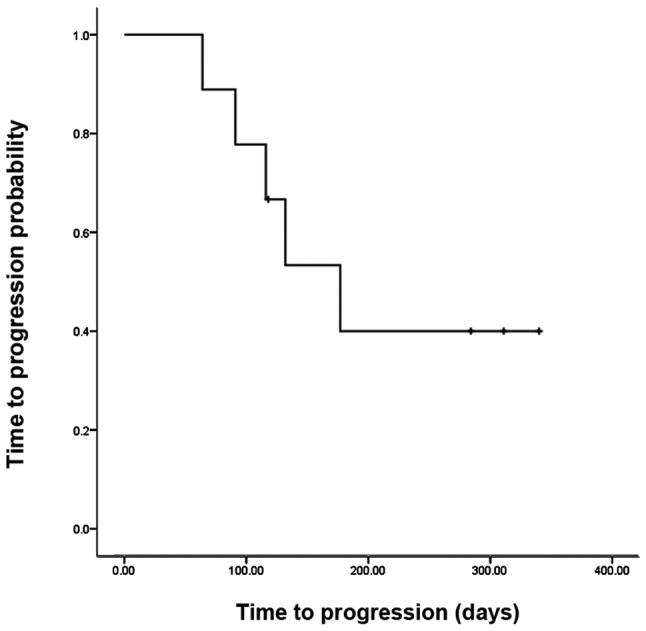
TTP for patients with NSCLC: The median TTP was 177 days, and the 6-month TTP rate was 40.0%. TTP, time to progression.

Although we conducted a phase I study, the follow-up time was not extensive, and the overall survival data are not yet available. However, we have reported the preliminary survival information in the current study. Fig. 1 indicates that the median TTP among all patients was 132 days, and the six month TTP rate was 39.2%. Fig. 2 indicates that among the NSCLC patients, the median TTP was 177 days (95% confidence interval: 100–255 days), and the 6-month TTP rate was 40%.

## Discussion

The current phase I dose escalation clinical trial demonstrated that the LBP and TXT combination regimen for the treatment of human solid tumours with progression following chemotherapy is safe, is associated with a low incidence of serious adverse effects, and exhibits short-term efficacy.

The wide application of DDP, carboplatin, and other platinum-based drugs in clinical antitumour therapies has led to the accumulation of overwhelming evidence indicating that platinum compounds have good therapeutic effects in the treatment of different types of cancer and that these compounds are currently among the most effective anticancer drugs in clinical use. However, as these drugs not only have significant renal toxicity and neurotoxicity, but also may cause auditory nerve damage as well as severe nausea and vomiting, the clinical applications of platinum-based drugs are subject to certain restrictions. Furthermore, the general physical condition and treatment tolerance are often poorer for patients receiving second-line therapy than for patients receiving first-line treatment. Therefore, current second-line treatment regimens frequently utilise drugs with lower toxicity and improved safety compared with platinum-based compounds.

DDP and the third-generation platinum compound, LBP have similar mechanisms of action. LBP has anti-cancer activity against a variety of tumours, including tumours resistant to DDP; LBP also produces only mild adverse gastrointestinal reactions, exhibits good water solubility, and has no significant nephrotoxicity or neurotoxicity (33,34). LBP was researched and developed by the German company ASTA Medica (Degussa) (5). In 2002, the Chinese firm Hainan Chang’an International Pharmaceutical Co., Ltd. purchased the LBP patent and exclusive LBP production and marketing rights. In 2005, the State Food and Drug Administration (SFDA) of China approved LBP and subsequently LBP entered the Chinese market as a new Class I drug that was predominantly utilised for the treatment of advanced breast cancer, SCLC, and chronic myelogenous leukaemia. In China, LBP is generally administered at doses of 30–50 mg/m^2^, with a 50 mg/m^2^ dose used for single-agent chemotherapy with LBP and a 30 mg/m^2^ dose used for combination therapies; LBP treatment is typically repeated every three to four weeks (5). However, the aforementioned doses have not been determined through rigorous phase I/II clinical trials. In Germany, phase I/II clinical trials of LBP were conducted on Western subjects; the findings from these trials were used to establish a recommended dose of 50 mg/m^2^ for single-agent LBP therapy (17). However, considering the different tolerances for the same doses of chemotherapy between Eastern and Western populations, it is unclear whether dose recommendations based on studies of Western subjects are applicable to Eastern patients. Furthermore, no prior phase I/II studies of LBP doses in combination regimens were identified. Certain foundational studies have suggested that LBP and TXT exhibit synergistic antitumour effects when used in combination (11) and that TXT is a broad-spectrum antitumour drug that can effectively treat a variety of malignant solid tumours (35). In addition, studies from East Asia have demonstrated that the administration of 60 mg/m^2^ of TXT to Eastern patients is as effective as and less toxic than the administration of 75–100 mg/m^2^ of TXT to Western patients (22–24). This phenomenon may be associated with to a lack of CYP3A (cytochrome P450, family 3, subfamily A) isoenzymes among Asian populations, given that these enzymes are involved in the metabolism of TXT to less active metabolites (22). Our previous studies have also found that the MTD for Chinese patients in a DDP and 5-fluorouracil combination regimen was equivalent to 70% of the corresponding MTD for Western patients (25). The aforementioned studies based on Asian populations have suggested that Eastern populations might have lower tolerances for doses of chemotherapy drugs than Western populations. Due to this, a phase I trial was conducted to identify MTDs in Eastern subjects; this clinical trial investigated LBP in combination with TXT to determine the MTD of LBP in this combination regimen.

Among the four cases of DLT in the current study, three cases of neutropenia were observed; by contrast, other studies have reported DLT occurring in the form of severe thrombocytopenia (17). In multiple clinical trials, the incidence of grade III–IV thrombocytopenia in LBP monotherapy (at 50 mg/m^2^) has ranged from 26.0%–72.7% (3,18,19). In studies of combination chemotherapy regimens involving LBP, typically at a dose of 30 mg/m^2^, the incidence of grade III–IV thrombocytopenia has ranged from 5.0%–23.8% (12,36); therefore, this incidence is markedly lower in combination chemotherapy compared with single-agent chemotherapy. Therefore, we considered the possibility that in the aforementioned studies, the incidence of chemotherapy-induced thrombocytopenia primarily correlated with the LBP dose. The 30 mg/m^2^ LBP, 35 mg/m^2^ LBP, and 40 mg/m^2^ LBP groups were established. The incidence of grade III–IV thrombocytopenia in the current study was 5.9% (1/17). The analysed LBP doses (30–40 mg/m^2^) were lower than the LBP dose used for single-agent chemotherapy (50 mg/m^2^), and the observed incidence of grade III–IV thrombocytopenia (5.9%) was also lower in our study compared with previous studies of LBP monotherapy (26.0%–72.7%) (3,18,19). In the current study, the observed case of grade III thrombocytopenia occurred in the 40 mg/m^2^ group, further indicating that the incidence of thrombocytopenia is associated with the LBP dose. In this study, the majority of observed toxicities were mild to moderate, and symptomatic treatment enabled a return to normal following the adverse events. Therefore, patients exhibited a favourable tolerance for the tested regimen.

In the current study, the RR was 7.1% (1/14), and the DCR was 78.6% (11/14). Among NSCLC patients, the RR was 11.1% (1/9), and the DCR was 77.8 % (7/9). However, He *et al* (37) reported that, for the second-line treatment of NSCLC with 30 mg/m^2^ LBP in combination with 75 mg/m^2^ TXT, an RR of 26.7% (4/15) and a DCR of 73.3% (11/15) were observed. Zhang *et al* (12) reported that, among patients with anthracycline-resistant advanced breast cancer who were treated with 30 mg/m^2^ LBP in combination with 75 mg/m^2^ TXT, an RR of 54.8% (23/42) and a DCR of 80.9% (34/42) were observed. The current study reported a lower RR than those reported previously (12,37). The following reasons may contribute to explaining this difference. Firstly, significantly higher treatment efficacy has been observed for the second-line treatment of breast cancer compared with that for the second-line treatment of NSCLC. The patients enrolled in the current study predominantly suffered from NSCLC, which was involved in 64.7% (11/17) of the cases that were examined. It was found that the efficacy of second-line chemotherapy for NSCLC was lower than that for breast cancer. However, Zhang *et al* (12) recruited patients with breast cancer and in the present study the majority of patients exhibited NSCLC. Thus, RR in the current study may have been lower than that reported by Zhang *et al* (12) due to patient differences. Secondly, this study included several patients who were receiving third-line NSCLC treatments. These patients accounted for 27% (3/11) of the examined cases of NSCLC. By contrast, the NSCLC study by He *et al* (37) involved only second-line treatment groups. The DCR calculated in the current study was consistent with the DCRs calculated in the aforementioned reports by Zhang *et al* (12) and He *et al* (37).

In the current study, the median TTP among NSCLC patients was 177 days (95% confidence interval: 74–163 days). In a review of second-line treatment for advanced NSCLC, Weiss *et al* (38) determined that the median TTP for second-line NSCLC treatment using cytotoxic chemotherapy agents ranged from 55 to 87 days and that the median TTP for second-line NSCLC treatment using epidermal growth factor receptor tyrosine kinase inhibitors varied from 48 to 108 days. The TTP in the current study was highly comparable with the findings by Weiss *et al* (38) in the review of second-line treatment for NSCLC; furthermore, 27% (3/11) of NSCLC patients in the current study were third-line patients. Therefore, with respect to both disease control rate and median TTP, these findings are encouraging.

In conclusion, the MTD for the examined LBP combination regimen was 35 mg/m^2^ LBP on d2 and 60 mg/m^2^ TXT on d1 of a treatment cycle that was repeated every 21 days. We are utilising these dosages in a prospective phase II study to further evaluate the efficacy and safety of the proposed MTD.

## Figures and Tables

**Table I:** Patient characteristics

Characteristic	Patients, n
Gender	
Male	9
Female	8
Age, year	
Range	45–76
Median	62
Stage	
IIIA	1
IIIB	2
IV	14
KPS	
Range	70–90
Median	90

**Table II:** Haematological toxicities.

	30 mg/m^2^		35 mg/m^2^		40 mg/m^2^	
			
	Cases, n	%	Cases, n	%	Cases, n	%
Leukopenia						
0	1	33.3	1	10.0	1	25.0
I–II	2	66.6	6	60.0	1	25.0
III	0	0	3	30.0	2	50.0
IV	0	0	0	0	0	0
Neutropenia						
0	3	100	3	30.0	1	25.0
I–II	0	0	2	20.0	1	25.0
III	0	0	4	40.0	1	25.0
IV	0	0	1	10.0	1	25.0
Anemia						
0	2	66.6	4	40.0	1	25.0
I–II	1	33.3	6	60.0	3	75.0
III	0	0	0	0	0	0
IV	0	0	0	0	0	0
Thrombocytopenia						
0	2	66.6	7	70.0	2	50.0
I–II	1	33.3	3	30.0	1	25.0
III	0	0	0	0	1	25.0
IV	0	0	0	0	0	0

**Table III:** Non-haematological toxicities.

	30 mg/m^2^		35 mg/m^2^		40 mg/m^2^	
			
	Cases, n	%	Cases, n	%	Cases, n	%
Diarrhea						
0	3	100	9	90.0	4	100
I–II	0	0	1	10.0	0	0
III–IV	0	0	0	0	0	0
Phlebitis						
0	3	100	9	90.0	4	100
I–II	0	0	1	10.0	0	0
III–IV	0	0	0	0	0	0
Fatigue						
0	3	100	6	60.0	3	75.0
I–II	0	0	4	40.0	1	25.0
III–IV	0	0	0	0	0	0
Nausea						
0	3	100	9	90.0	2	50.0
I–II	0	0	1	10.0	2	50.0
III–IV	0	0	0	0	0	0
Vomiting						
0	3	100	10	100	4	100
I–II	0	0	0	0	0	0
III–IV	0	0	0	0	0	0

**Table IV:** Dose escalation level.

Levels	Patients	Lobaplatin, mg/m^2^	Docetaxel, mg/m^2^
I	3	30	60
II	10	35	60
III	4	40	60
